# Study protocol for a randomised controlled trial to evaluate the effectiveness of a serious game targeting interpersonal emotion regulation in early adolescents

**DOI:** 10.1186/s13063-021-05706-7

**Published:** 2021-10-25

**Authors:** Gloria Mittmann, Sonja Zehetmayer, Beate Schrank

**Affiliations:** 1 D.O.T. Research Group for Mental Health of Children and Adolescents, Ludwig Boltzmann Society at Karl Landsteiner University of Health Sciences, Krems, Austria; 2 Department of Developmental and Educational Psychology, University of Vienna, Vienna, Austria; 3 Center for Medical Statistics, Informatics, and Intelligent Systems, Medical University of Vienna, Vienna, Austria; 4 Department of Psychiatry and Psychotherapy, University Hospital Tulln, Karl Landsteiner University of Health Sciences, Tulln, Austria

**Keywords:** Interpersonal emotion regulation, Extrinsic emotion regulation, Serious games, Sex differences, Gender differences, Early adolescence, Preadolescence, Social connectedness, Friendship

## Abstract

**Background:**

Adaptive interpersonal emotion regulation (iER) is a vital tool for positive relations. During early adolescence, peer relations become increasingly important, making this age group a relevant target group for interventions promoting positive interactions with each other, yet no evidence-based intervention exists for iER and early adolescents specifically.

**Methods:**

This randomised controlled trial (RCT) aims to test effectiveness and feasibility of a serious game training iER skills in early adolescents by comparing outcomes with a control group playing a game without psychoeducational content in a pre- and post-test design. German- and English-speaking early adolescents (10–14 years) are eligible for participation. IER skills improvement as assessed by a vignette task is the primary outcome and will be analysed with a chi-square test. Secondary outcomes include feasibility and acceptability, emotional competence, personal emotion regulation, gender, and sex.

**Discussion:**

This RCT will test whether playing a serious game about iER strategies results in an improvement of iER skills and whether the game is feasible and acceptable for early adolescents with the ultimate aim to implement the game in schools and help early adolescents achieve positive peer relationships.

**Trial registration:**

ClinicalTrials.govNCT04808102, Registered on 19 March 2021

## Administrative information

Note: the numbers in curly brackets in this protocol refer to SPIRIT checklist item numbers. The order of the items has been modified to group similar items (see http://www.equator-network.org/reporting-guidelines/spirit-2013-statement-defining-standard-protocol-items-for-clinical-trials/).

**Table:** 

Title {1}	Study protocol for a randomized controlled trial to evaluate the effectiveness of a serious game targeting interpersonal emotion regulation in early adolescents
Trial registration {2a and 2b}.	ClinicalTrials.gov, NCT04808102, Registered 19 March 2021, https://register.clinicaltrials.gov/
Protocol version {3}	Protocol version 1.0 (March 19, 2021)
Funding {4}	This work is supported by the Ludwig Boltzmann Society and the Karl-Landsteiner University of Health Sciences
Author details {5a}	GM – D.O.T. Research Group for Mental Health of Children and Adolescents, Ludwig Boltzmann Society at Karl Landsteiner University of Health Sciences, Krems, Austria, gloria.mittmann@kl.ac.at SZ – Center for Medical Statistics, Informatics, and Intelligent Systems, Medical University of Vienna, Vienna, Austria, sonja.zehetmayer@meduniwien.ac.at BS - Department of Psychiatry and Psychotherapy, University Hospital Tulln, Karl Landsteiner University of Health Sciences, Tulln, Austria, beate.schrank@kl.ac.at
Name and contact information for the trial sponsor {5b}	Ludwig Boltzmann Society GmbH, Nußdorfer Str. 64, 1090 Wien
Role of sponsor {5c}	The funding body and study sponsor have no role in the design of the study, analysis, or interpretation of the data

## Introduction

### Background and rationale {6a}

#### Interpersonal emotion regulation

Since the development of the process model of emotion regulation (ER) [1], numerous studies have investigated the ways and effects of ER [2, 3]. In the last decades, the concept of interpersonal ER (iER) has developed out of the concept of ER, but even though research about iER is on the rise, it has still received substantially less attention. In general, iER can be defined as deliberately influencing the emotions of another person [4]. Niven, Totterdell [4] found that iER can either improve or worsen affect. Improving others’ emotions can be achieved in two main ways: either dealing with the situation/emotion directly (positive engagement) or by focusing on the person experiencing the emotion without engaging with the emotion directly (acceptance, see Table 1). This classification has been validated by López-Pérez, Morillo [5] and will therefore be used in this protocol and study.

**Table 1:** Classification of adaptive iER by Niven, Totterdell [4]

Cluster	Positive engagement		Acceptance	
Sub-cluster	Affective engagement	Cognitive engagement	Humour	Attention
Definition	Improve way the target feels about a situation	Change way the target thinks about a situation	Be humorous to communicate validation	Give the target attention to communicate validation
Example	Listen to the target’s problems	Give the target advice	Joke with the target	Make clear you care about the target

Henceforth, we will use the terms “personal ER (pER)” for regulating one’s own emotions and “interpersonal ER (iER)” for regulating someone else’s emotions to clearly distinguish between the two concepts.

#### Sex differences in iER

Sex differences have been found in pER [6, 7], but data is lacking when it comes to iER specifically. In the general population, females seem to have better interpersonal skills than males, while males score higher on intrapersonal skills [8] and studies about related concepts such as emotional intelligence [9], emotional reliance [10], or coping [11] found sex differences.

To our knowledge, López-Pérez, Morillo [5] are the only ones who tested sex differences concerning specific iER strategies defined by classifications. They found that women tend to use positive engagement more often than men, which gives preliminary evidence that women engage in the social affective component of iER strategies more than men. In their study, age varied from 18 to 76, thus excluding the targeted group of our study. There might be stronger differences during adolescence when sexuality, and therefore also gender, starts to be an important topic [12].

#### Early adolescents and iER

One age group that might especially benefit from high iER skills are early adolescents (broadly defined as 10–14 years, see [13]). Due to rapid developmental and psychosocial advances [14], the relationship with peers becomes increasingly important during that time. They not only influence externalising behaviour such as alcohol and drug use [15] or risk behaviour [16] and academic achievement [17], poor peer relations are also related to social anxiety and depression [18]. Consequently, positive peer relations are a vital buffer for coping with stressors and maintaining good mental health during early adolescence.

IER is an important skill for successful communication and interaction. This includes, for example, communication skills, which are important for the perceived quality of a friendship [19]. Using iER strategies can improve feelings of trust and friendship with peers [20] and helps finding new friends [21].

Despite the importance of interpersonal relationships during early adolescence, existing studies mostly concentrate on pER. Findings from that domain suggest that pER has an influence on disordered eating [22], sexual behaviour [23], or mental health symptoms like depression [24].

#### Serious games

It has been shown that analogue intervention and prevention programs in general have the potential to improve social emotional skills and wellbeing [25], but furthermore, both research and industry have started to pay attention to the advantages of the digital settings of serious games [26] as a means of intervention and learning. Serious games can be broadly defined as games that have an aim surpassing simple entertainment [27].

In a meta-analysis, Wouters, van Nimwegen [28] found that teaching cognitive skills through serious games was more effective than conventional methods. A literature review by Hamari, Koivisto [29] and a meta-analysis by Sitzmann [30] also found that gamification can work. Yet, findings of positive effects are still weak due to meagre literature [31], underlining the need for rigorous testing of serious games. One important advantage of serious games is that they boost players’ motivation [29]. As gaming is a rapidly growing industry that adolescents deem highly entertaining and important [32], serious gaming can be a potent tool to engage early adolescents in a fun way to learn about social skills.

Most existing serious games on ER have a focus on pER [33, 34], but some have also included interpersonal components. For example, in SPARX [35], depressed adolescents learn both personal and interpersonal strategies (such as communication and negotiation skills), but to our knowledge, only one game has specifically focused on iER. López-Pérez and Pacella [36] examined iER in children (8–10 years) with the use of a serious game called Emodiscovery [37]. This game lets children use different adaptive and maladaptive strategies to handle various scenarios where someone needs to be cheered up.

### Limitations and rationale

Overall, there are some gaps and limitations in the existing literature that lead to the idea and necessity of this study, most importantly the lesser focus of research on iER and the lack of serious games for a general population of early adolescents.

Concluding, iER skills can influence the successful establishment and maintenance of friendships and relationships of early adolescents, yet literature on iER in that age group is scarce. Research thus far has not sufficiently understood the concept of iER and the potential of training it targeting adolescents on a broad scale in the general public. We aim to fill this gap by developing a serious game that helps early adolescents express their strategies in situations requiring iER skills. The development of the game included an extensive literature search on iER, and in-game strategy-options were generated by using original material from Niven, Totterdell [4] study, which the authors kindly provided. Co-development workshops with children of the relevant age group helped with including ideas and remarks from the target group. The interdisciplinary research team included a diverse range of professions such as psychologists, computer scientists, and playwrights.

### Objectives {7}

In this RCT, we will assess the impact of the web-based serious game on iER skills in early adolescents. The primary objective of this study is to compare the improvement in pre- and post-intervention knowledge about iER strategies between a group that plays the intervention/serious game and a control group that plays a game without psychoeducational content. The secondary objectives are to examine sex differences in the use of specific strategies and to evaluate feasibility and acceptability of the intervention. We will examine the effects on outcomes of the following potential confounding variables: gender, prior gaming experience, language, and pER.

### Research questions

1. *Effectiveness*: How effective is playing the iER adventure game (AG) compared to a control group in terms of skills and knowledge regarding emotions and iER?
2. *Sex differences*: Do boys and girls prefer different specific iER strategies according to Niven et al. (2009)’s classification?
3. *Feasibility*: Is the iER AG feasible and acceptable in the targeted population?

### Hypotheses

1. *Emotional skills*: Participants of the experimental group will be able to recall more adaptive iER strategies after playing the iER AG compared to the control group.
2. *Emotional skills*: Participants of the experimental group will score higher on the Emotional Skills and Competence Questionnaire (ESCQ; Takšić et al., 2009) after the iER AG compared to baseline pre-test scores and compared to the control group.
3. *Emotional skills*: Participants of the experimental group will score higher on the Emotion Regulation of Others and Self scale (EROS; Niven et al., 2011) after the iER AG compared to baseline pre-test scores and compared to the control group.
4. *Sex differences*: In a computer animated game environment which requires players to use iER strategies in social interactions, male and female participants will prefer different iER strategies. Female participants are more likely to engage in positive engagement, whereas male participants are more likely to engage in acceptance.

### Trial design {8}

This trial is a RCT with an experimental and a control group, with participants being individually allocated to either of the groups. The allocation ratio is 50:50, and the framework is exploratory.

## Methods: participants, interventions, and outcomes

### Study setting {9}

This study will be conducted online and is consequently not linked to a specific country, but recruitment will take place primarily in German- and English-speaking countries with the main countries being Austria and the UK.

### Eligibility criteria {10}

Eligible participants will be early adolescents between 10 and 14 years. Furthermore, participants have to be able to read and understand German or English as the game can be played in these two languages. They must also provide written parental consent and be willing to participate in the study by filling out questionnaires at two time-points (pre- and post-test) and playing a web-based game in between. The game targets iER in the general population of early adolescents. Hence there will be no restrictions in terms of learning difficulties or mental health problems (see Table 2).

**Table 2:** Eligibility criteria

	Inclusion criteria	
1.	Between 10 and 14 years old	
2.	Willing and able to provide written parental consent	
3.	Willing and able to participate in filling out pre- and post-test online questionnaires and playing a web-based game	
4.	Able to read and understand German or English	
5.	Access to a device with internet (computer, mobile phone, tablet)	

### Who will take informed consent? {26a}

Parents/legal guardians of eligible adolescents have to confirm their consent via a pre-scripted email sent to the research group. The lead investigator (GM) will accept the emails. Links to the study questionnaires are only sent after receiving this email. Adolescents will tick a box at the beginning of the pre-test questionnaires asking for their consent.

### Additional consent provisions for collection and use of participant data and biological specimens {26b}

This is not applicable as this is not an ancillary study.

## Interventions

### Explanation for the choice of comparators {6b}

This is an early stage first analysis of the developed game. We are interested in the effects of the whole game, not its specific components. Therefore, we use a puzzle game without psychoeducational components as comparator to control for time spent online with a game. Using another game instead of no game at all helps consider expectation bias and potential lack of interest to continue the study in a control group.

## Intervention description {11a}

### Experimental intervention

An iER adventure game was/is currently being designed and developed using both scientific research and co-development workshops with children of the targeted age group to assure that the game has both learning and fun components. It is a single-player role-play/adventure game with 3 levels and a hub world (connecting the levels). It is played from a top-down perspective and created with the commercially available RPG Maker MV. The game is played on a phone or a browser. The game software will allow us to collect data on how the game was played, i.e. which options the participants chose and different time points (e.g. start time, end time). All conversations in the game will be presented in text through conversation windows. Total playtime depends on the player, as the explorative nature of the game allows more or less exploring, but to complete the game, approximately 3 h will be necessary. For the duration of the study, the game will be freely accessible (but will ask for a participant code to enter) via https://dot-game.picapipe.dev/.

#### Storyline

The setting of the game is in a school, the playable character (PC) is a pupil. The game starts with a normal school day for the PC to familiarise the player with the style of the game. After that day, the PC stays home for a day because they are sick. They receive strange, rude text messages during this time. When they return to school, everything has changed. Pupils are very aggressive, fighting and arguing everywhere. Suddenly, there is an explosion, and everyone is gone. The school is dark and dusty. The PC does not know what happened. While exploring, the PC finds four other remaining pupils and forms a group of friends with them. Together, they pass through three portals that can be found in the school and that lead to different fantasy worlds. In these fantasy worlds, the group needs to find their way back and save the pupils that have vanished. Between levels, the group meets a mysterious goldfish who turns out to be the guardian animal of the school. The goldfish helps them understand the interpersonal conflicts, the reason for the catastrophe, and their role in resolving it. After completing each fantasy world, the school is restored a little bit more. After completing all worlds (levels), the school is fully restored. Nobody except the group remembers what happened.

#### Psychoeducational component

The game teaches and trains iER through various techniques:

1. Interaction with non-playable characters (NPCs): The group encounters a variety of NPCs on their way who show various emotions. The player needs to interact with these NPCs appropriately and use adaptive strategies to help them deal with their emotions. Emotions include fear, anger, disappointment, and sadness.

2. Interactions within the group: The player receives information about group members during conversations and gameplay that need to be remembered and addressed appropriately. This can for example be information about fears (someone is scared of the dark).

3. Happy, the goldfish: The player meets the goldfish after each level. It serves to help internalise and repeat psychoeducational content learned during the level.

#### Feedback

The teaching of correct iER strategies is mainly achieved by varying feedback. Ultimately, the player cannot proceed and finish the game when using the wrong (i.e. maladaptive) strategies. Specifically, there will be:

- Audio and visual feedback: sounds and pictures (similar to emojis, e.g. heart-shaped) over the receiving agent’s head will indicate if a strategy was successful.
- Feedback from receiving agents: Receiving agents will clarify if a strategy was good (e.g. “Thank you for listening to me without interrupting!”) or maladaptive in this situation (e.g. “That just made me feel worse.”).
- Rewards and achievements: After some successful interactions, the player gets rewarded with items (received as gifts from the receiving agent). Items can be sold for in-game money or are needed in further interactions or levels to proceed in the game.

### The control intervention

The control group will receive a free-to-play arcade browser puzzle game with no psychoeducational content. The game requires the player to clear a field of multicoloured bubbles by trying to group same-coloured bubbles into groups of three or more. Single coloured bubbles are used one by one to shoot at the field and change its configuration. The game is won when no bubble remains and can then be started again. It is freely available under https://www.squidbyte.com/games_third/bubbleshooter/bubble-shooter/.

### Criteria for discontinuing or modifying allocated interventions {11b}

Neither the measurements nor the gameplay is expected to result in any significant harmful side-effects, as the game has no graphic depiction of violence and none of our measures are invasive. Thus, we do not expect that we need to modify the interventions during the duration of the study. As the study is completely automated and anonymous, individual participants cannot be identified.

### Strategies to improve adherence to interventions {11c}

This study is executed online. Researchers have little influence after participants begin the study, but deviation from the protocol is unlikely due to automated online processes.

### Relevant concomitant care permitted or prohibited during the trial {11d}

There will be careful consideration to avoid active recruitment of participants that have already participated in co-development workshops e.g. by not advertising the study in schools that have recently been or currently are part of another study or workshop with our research group. Schools that participated more than a year ago pose no problem as pupils from classes that participated would be excluded due to the narrow age span of the study.

### Provisions for post-trial care {30}

Neither the measurements nor the gameplay is expected to result in any significant side-effects, as the game has no graphic depiction of violence and none of our measures are invasive.

### Outcomes {12}

Our primary outcome in this study is the change of iER strategies participants can think of from pre (baseline)- to post-intervention, i.e. if the percentage of participants who improve their knowledge about possible iER strategies is higher in the experimental group compared to the control group. This is done via a vignette-task. The follow-up will be approximately 5 days after baseline measures.

In secondary analyses, we will test iER as a corroborating variable with a questionnaire. The questionnaire was not developed to measure change of iER skills over a short period of time (it asks for iER strategies use in the last 2 weeks) and is thus used as a complementary measure. As the game addresses general topics around emotions additionally to specific iER strategies, we will also examine the change in general emotional competence. Furthermore, effects of sex and gender on strategy choice and feasibility of the game will be examined.

### Participant timeline {13}

This RCT adopts a pre-post-test strategy with the between-participant factor “group” and the within-participant factor “time”. Assessment will include pre-tests (questionnaires; day 1), intervention (playing the game; days 1–5), and post-tests (questionnaires and acceptability measures; days 5–10). After sending the consent forms, every participant will receive the link to the pre-test questionnaires via email. They take approximately  30 minutes to complete. At the end of these questionnaires, participants will be randomly assigned to either experimental or control group and receive the appropriate link for the corresponding game. Additionally, participants are asked to provide a unique identifying code consisting of the first letter of their own first name, the first letter of their mother’s first name, the first letter of their father’s first name, and the day of their birthday. They will use this code in the subsequent parts of the study to allow to link the parts anonymously. The link for the games can be opened as soon as they receive it and accessed with the unique identifying code that they used in the pre-test questionnaires already. Participants are asked to play the game for as long as they want but with the aim to finish it. After that, the participants have 5 days to play the game. They receive the link for post-test questionnaires 5 days after the first link was sent. The post-test questionnaires will take approximately 30 minutes to complete. After 2 days, a generalised reminder is sent to each participant via email to remind them to finish the study if they have not done so already.

**Fig. 1:**
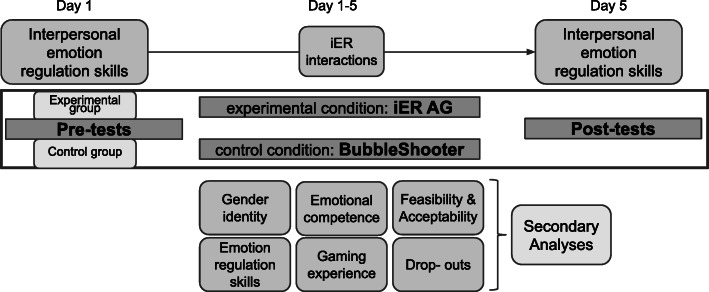
Study design for an RCT examining the effectiveness of a serious game targeting interpersonal emotion regulation

The iER AG allows for great variability in playtime, but no matter how long each player takes to finish the game, everyone will have the same amount of iER interactions. As long as they play the game to the end, playtime has no influence on the psychoeducational component but only on the explorative “fun” part of the game. In general, we expect the average playtime to be approximately 3 h in total. The control group will get instructions to play the game as much as they want for the next days. They will receive the final questionnaires 5 days after they started the study. For an overview of the study design, see Fig. 1.

### Sample size {14}

For the sample size calculation, we consider the percentage of adolescents with a positive change in iER skills between pre- and post-test. We assume that in the experimental group, the number of positive strategies improves by 30%, in the control group it improves by 10%. A chi-square test with a sample size of 62 adolescents per group will have 80% power (significance level 0.05). We expect approximately 30% drop-outs; thus, the total sample size is increased to 180. If in the control group only 1% improves compared to 21% in the experimental group, with this sample size the study has a power of more than 90%.

### Recruitment {15}

Participation is allowed for all people who comply with the inclusion criteria. This study will be conducted online and is consequently not linked to a specific country, but recruitment will take place primarily in German- and English-speaking countries. Given the strict age restriction, a special focus will be put on recruitment from secondary schools. We will actively approach schools in Austria and the UK (this is where the research team is located) and advertise the study via leaflets (paper and email), media presence, and teacher training events. Schools will be likely to participate due to prior and ongoing close collaboration with the research team. Furthermore, the project will be advertised online via social media. Interested participants and/or their parents will receive further information about the study and consent forms via email.

## Assignment of interventions: allocation

### Sequence generation {16a}

After receiving the consent of a parent or legal guardian, the participant will receive a link to the pre-test questionnaires. At the end of the pre-test-questionnaires, participants will be randomised into either experimental or control group. We use SoSci Survey for administering the questionnaires via www.soscisurvey.de [38]. This software allows automated randomisation into different groups. We will use the option to “equally distribute” participants to avoid different group sizes. Depending on the group, participants will then receive the link to either intervention or control game directly in the questionnaire. Participants as well as researchers are blinded during the whole trial.

### Concealment mechanism {16b}

As the allocation is done during questionnaires by the software SoSci Survey, sequences are unknown to both researchers and participants.

### Implementation {16c}

Both generation of the allocation sequence and assignment to the intervention groups is executed by a computer system. Participants are enrolled by the lead investigator (GM), who does not know the allocation sequence.

## Assignment of interventions: blinding

### Who will be blinded {17a}

Trial participants are blinded throughout the study. Statistical analysis will be executed by generating a number for each group, with its affiliation unknown to the analyst.

### Procedure for unblinding if needed {17b}

Due to anonymization, unblinding is not possible. Researchers have minimal contact with participants during the trial and potential harms are unlikely due to the non-invasive psychological nature of the trial.

## Data collection and management

### Plans for assessment and collection of outcomes {18a}

#### Primary outcome—interpersonal emotion regulation

Vignettes [39] are used to depict short sequences of events and have been successfully used in psychological research (e.g. [40]). While often consisting of short written text, we will use visual vignettes [41] (to reduce reading for the participants and to be able to show emotional expressions) with hypothetical situations that require the use of iER before and after the game to assess if children’s perception of their options have changed, i.e. if they can think of more strategies to help a person in a hypothetical situation after they have played the iER AG. A visual scene will depict an every-day life situation with a person experiencing a negative emotion that needs to be regulated. Every participant will receive the same four different scenarios, containing 1 image depicting the scene (Table 3). For each scenario, participants get an open-ended question (“If you were in that room, what could you do in this situation to help this person? Write down everything you can think of.”).

**Table 3:** Vignette task for interpersonal emotion regulation strategies

	Scenario	Emotion
1.	An early adolescent trips over in front of their peers.	Embarrassment
2.	An early adolescent is scared of a spider.	Fear
3.	An early adolescent is being excluded by their peers.	Sadness
4.	An early adolescent is angry because someone broke their phone.	Anger

#### Secondary outcomes

*Emotion regulation skills*. The Emotion Regulation of Others and Self scale (EROS [42];) will be used to measure both personal and interpersonal ER. This scale includes four factors of ER: intrinsic (personal) affect-improving, intrinsic affect-worsening, extrinsic (interpersonal) affect-improving, and extrinsic affect-worsening and shows good internal reliability and construct validity. Of the overall 19 items, 9 measure extrinsic (interpersonal) ER and 6 extrinsic affect-improving ER. The instructions ask to report the extent the person had used the strategy over the past 4 weeks to try to change their own or someone else’s feeling on a five-point Likert scale (“not at all”, “just a little”, “moderate amount”, “quite a lot”, “a great deal”). One example item is: “I listened to someone’s problem.”. The mean score in an adult population for extrinsic affect-improving is 3.53, for extrinsic affect-worsening 1.20. Internal consistency ranges from *α* = 0.74 to *α* = 0.82. The measure will be translated into German according to standard recommendations for the translation and adaptations of questionnaires [43].

*Emotional competence*. To measure emotional competence in early adolescents, the Emotional Skills and Competence Questionnaire (ESCQ [44];) will be used. The questionnaire consists of 45 items on three subscales: the Perceive and Understand emotions scale (15 items; e.g. “When I see how someone feels, I usually know what has happened to him”), the Express and Label emotions scale (4 items; e.g. “I am able to express my emotions well.”), and the Manage and Regulate emotions scale (16 items; e. g. “When I am in a good mood, every problem seems soluble”). Ratings are given on a 5-point scale (“never” to “always”). Internal consistency ranged from α = .81 to .90 (Perception and Understanding), .78 to .88 (Express and Lable), and .67 to .78 (Manage and Regulate), and .88 to .92 (overall emotional competence). The measure will be translated into German by the same procedure as described before.

*Sex differences*. Sex differences will be examined in the experimental subgroup of our sample. During gameplay, the player undergoes 10 different scenarios, where they interact with NPCs by choosing one of up to four dialogue options. The four different strategies according to Niven, Totterdell [4] will be presented during these interactions. The dialogue options will consist of two adaptive and two maladaptive strategies (on average, with a range of 2–4 adaptive options, but always including both strategies of “positive engagement” and “acceptance”). Each picked option will be saved by the game, compiling an option pattern for each participant.

*Feasibility and acceptability*. For measuring feasibility, we will use the short version of the Game User Experience Satisfaction scale (GUESS-18 [45];). The scale has 18 items on a 7-point Likert scale (“strongly disagree” to “strongly agree”). It has 9 subscales (with two items each) covering aspects of gaming: usability/playability, narratives, play engrossment, enjoyment, creative freedom, audio aesthetics, personal gratification, social connectivity, and visual aesthetics. We will exclude the subscale “social connectivity” as the game is not a multiplayer game. Scores of different subscales can be added for an overall score. First validity measures show excellent convergent validity, with all subscales above 0.722. Reliability is *α* = .785 for the overall scale. In addition to the GUESS-18, we will analyse the time that was spent on the game and perform sensitivity analyses for drop-outs. The measure will be translated into German by the same procedure as described before.

*Gender*. Gender (in contrast to sex) will be assessed with the Inventar zur Erfassung des Geschlechtsrollen-Selbstkonzepts im Jugendalter (GRI-JUG [46];). This instrument assesses gender identity on four subscales: positive and negative feminine (communal; e.g. “emotional”, “vengeful”) and masculine (agentic; “companionable”, “lazy”) attributes. Each of the subscales consists of 5 items, yielding a total of 20 items. Subjects are asked to rate how much the attributes relate to themselves on a 5-point Likert scale (“does not apply at all” to “applies (nearly) always”). Internal consistencies of the scales are α = .81 (Mas+), *α* = .80 (Mas−), *α* = .88 (Fem+), and *α* = .74 (Fem−). Official English translation of the items exists.

*Gaming experience and preference*. Willingness to engage and hence outcomes might be influenced by gaming experience and gaming preference [47]. The style of the game in this study might remind some participants of games they already played as it is a common style in especially older AGs and some participants might have more experience. We will cover gaming experience with four questions specific to the current study: [1] Do you like videogames (answers are given on a 4-point scale: yes; rather yes, rather no, no)? [2] How much do you play per week (not at all (0 h); little (0–2 h); medium (3–5 h), a lot (more than 5 h))? [3] Have you ever played an adventure game (yes; no)? [4] If yes, what was the name (open question)?.

*Sociodemographic questions and questions to assess potential contamination*. Sex and age will be asked at the beginning of the questionnaires. After the intervention, participants will be shown a screenshot of the intervention game and asked if they have seen this game before. As we cannot gather data of the control game, we will additionally ask for how long the game was played approximately (0-30 min; 30 min–1 h; 1–2 h; more than 2 h). For an overview of the measures, see Table 4.

**Table 4:** Measures used in the study

Measure	Assessment tool	Timepoint		
		Pre-test	In-game	Post-test
IER	Vignettes with hypothetical iER situations	x		x
ER	Emotion Regulation of Others and Self scale (EROS; 42)	x		x
Emotional competence	Emotional Skills and Competence Questionnaire (ESCQ; 44)	x		x
IER strategies	In-game option patterns		x	
Gaming experience	Self-developed questions	x		
Gender	Inventar zur Erfassung des Geschlechtsrollen-Selbstkonzepts im Jugendalter (GRI-JUG; 46)	x		
Acceptability	Game User Experience Satisfaction scale (GUESS-18; 45). In-game measures (drop-outs, time)		x	x

### Plans to promote participant retention and complete follow-up {18b}

Participants are encouraged to finish the study by raffling 5 Amazon vouchers for 50 Euros between all participants who finish the study. Participants have to enter their email addresses at the end of post-test questionnaires. SoSci Survey allows to handle email addresses for this process independent of the rest of the study to ensure anonymity. Furthermore, a standardised email will be sent to every participant 2 days after sending the post-questionnaire link to remind participants to finish the study if they have not done so already.

### Data management {19}

All measures will be administered via online questionnaires at both pre- and post-test via www.soscisurvey.de [38]. This German server is ISO27001 certified and guarantees organisational, structural, and technical security in accordance with current standards. The server is accessed via SSH-encrypted connection. No data is passed on to third parties. No other person has access to the data, in accordance with section 11 of the Federal Data Protection Act. The server uses SSL encryption. Data is already encrypted on the participant’s browser and only decrypted on the server. No IP addresses are stored in the access log files and no cookies are used.

Due to questionnaires being administered online, missing values can be prohibited by not allowing to skip questions. Outcome data will be checked for unrealistic answer patterns (e.g. always the first option).

Outcome data will be stored on secure, password-protected platforms at the Karl Landsteiner University of Health Sciences. Only direct research associates can access the data.

### Confidentiality {27}

All data used in this study is anonymized. Email addresses will only be used to send out consent forms and links for the study but cannot be connected to the participants. Each participant will receive a unique identification number at the end of the pre-test questionnaires that they will use to log into the game and in all subsequent study parts. This code will be changed to one number for each participant for analysis as well as any data sharing.

### Plans for collection, laboratory evaluation, and storage of biological specimens for genetic or molecular analysis in this trial/future use {33}

Not applicable as this is neither an ancillary study nor includes the collection of biological specimens.

## Statistical methods

### Statistical methods for primary and secondary outcomes {20a}

#### Randomisation

To evaluate the randomisation process, baseline variables will be compared between experimental group and control group, first based on the intention-to-treat principle, which means that all participants are considered in the analyses regardless if they completed all questionnaires or not. Second, the baseline comparison will be repeated for the main analysis sample, which comprises all participants with completed questionnaires.

#### Primary analysis—interpersonal emotion regulation skills

First, descriptive statistics will be generated for each observation time (pre- and post) and for each group: for continuous variables, mean and standard deviation (or median and interquartile range in case of a skewed distribution, respectively) and for categorical variables absolute and relative frequencies will be computed.

Answers of the vignette task will first be qualitatively assessed to exclude maladaptive as well as nonsensical strategies and merge answers that can be defined to be the same strategy. To analyse the main question, the change in the absolute number of positive strategies between pre- and post-test as well as the percentage of adolescents with a positive change in iER skills will be considered. A chi-square test (main analysis) will be calculated to compare the percentage between the two groups. Then, a logistic regression analysis will be performed for the dependent variable “positive change yes/no” to investigate the influence of the independent variables “time between last time of playtime and post-test”, “language”, “total playtime”, and “group”. To compare the change in absolute numbers, a non-parametric Wilcoxon rank-sum test will be performed (secondary analysis). The significance level will be set to 0.05.

#### Secondary analyses

*Emotion regulation and emotional competence*. For the two questionnaires about emotion regulation and emotional competence, comparisons between groups will be performed for the pre-test and for the change between pre- and post-test (items change in the Likert scale is set to + 1 or − 1 if the Likert scale improves or worsens, by one point, respectively, and so forth). For both analyses, a Wilcoxon test will be performed.

*Sex differences within the experimental group*. Adaptive iER strategies will be dichotomised into positive engagement vs acceptance and for each participant the more frequent strategy will be determined. Our outcome variable is the proportion of which sex uses positive engagement (or acceptance). A chi-square test will be calculated to compare the percentage between the sexes. Second, a logistic regression analysis will be performed to evaluate if gender stereotype scores have an influence on the more frequent strategy positive engagement or acceptance. Note that for this analysis only adolescents from the experimental group will be considered.

*Feasibility*. Data from the feasibility questionnaire will be analysed using descriptive statistics. Open questions will be analysed qualitatively following thematic analysis [48].

For the secondary and exploratory analyses, the significance level for all tests is set to 0.05, *p*-values are interpreted in a descriptive manner. The statistical software programs R and SPSS will be used [49, 50].

### Interim analyses {21b}

We do not expect the trial to result in any harmful side effects, and due to online administration, the process will not be monitored. Yet, after an internal pilot phase of 40 participants, we will look at drop-out rates and—if patterns are found during questionnaire completion—possibly change the settings of the online questionnaires to allow participants to skip questions and increase adherence.

### Methods for additional analyses (e.g. subgroup analyses) {20b}

The additional analyses such as subgroup analyses are already covered by the secondary outcome analyses.

### Methods in analysis to handle protocol non-adherence and any statistical methods to handle missing data {20c}

Missing values will be avoided by not allowing participants to skip questions. Drop-outs will be considered in sensitivity analyses based on the intention-to-treat principle, which means that all participants are considered in the analyses regardless if they completed all questionnaires or not. No data imputation for missing data will be performed.

### Plans to give access to the full protocol, participant level-data and statistical code {31c}

We will deposit anonymised research data in an online repository of the Open Science Framework (OSF; https://osf.io/). OSF supports open, centralised workflows including developing a research idea, designing a study, storing and analysing collected data, and writing and publishing reports or papers.

## Oversight and monitoring

### Composition of the coordinating centre and trial steering committee {5d}

The current study does not have a coordinating centre or trial steering committee as the study is of psychological nature bearing negligible risks of harmful side effects and the whole procedure is fully automated and online.

### Composition of the data monitoring committee, its role and reporting structure {21a}

The current study does not have a data monitoring committee (DMC) as the study is of psychological nature bearing negligible risks of harmful side effects and the whole procedure is fully automated and online.

### Adverse event reporting and harms {22}

This study is completely online without direct contact between researchers and participant during participation.

### Frequency and plans for auditing trial conduct {23}

This study is completely online without direct contact between researchers and participant during participation.

### Plans for communicating important protocol amendments to relevant parties (e.g. trial participants, ethical committees) {25}

Amendments and changes will be transparently described in the publications following the trial.

### Dissemination plans {31a}

The results of the research will be published in peer-reviewed journals of general and special interest and presented at international conferences. Summaries of the results will be presented for the general population on the research group webpage (https://dot.lbg.ac.at/).

## Discussion

This study-protocol describes an RCT to examine the effectiveness of a serious game targeting iER in early adolescents. The ultimate goal is to include this game in the curriculum of Austrian schools as part of a larger complex prevention program to endorse social connectedness in peers who just underwent transition from primary to secondary school. Transition is a major changeover period that can have short- and long-term negative effects on children who do not manage to connect to their new peers [51, 52]. Thus, prevention programs are necessary and important during transition phases to help early adolescents with skills to form friendships [53]. After the evaluation of the individual components, an evaluation of the complete complex prevention program that includes the presented game of this protocol as well as other digital and analogue components endorsing competences such as pER, collaboration skills, or meta-cognition is planned.

## Trial status

Protocol version 1.0 (March 19, 2021). The trial status is currently in the pre-recruitment phase. Recruitment is planned to start in April 2020. Recruitment and trial are expected to be completed by October 2020.

## Abbreviations

AG: Adventure game
DMC: Data monitoring committee
ER: Emotion regulation
EROS: Emotion Regulation of Others and Self scale
ESCQ: Emotional Skills and Competence Questionnaire
GRI-JUG: Inventar zur Erfassung des Geschlechtsrollen-Selbstkonzepts im Jugendalter
GUESS-18: Game User Experience Satisfaction scale
IER: Interpersonal emotion regulation
NPC: Non-playable character
OSF: Open Science Framework
PC: Playable character
PER: Personal emotion regulation
RCT: Randomised controlled trial
